# Validation of Reference Genes for Gene Expression Studies in Virus-Infected *Nicotiana benthamiana* Using Quantitative Real-Time PCR

**DOI:** 10.1371/journal.pone.0046451

**Published:** 2012-09-28

**Authors:** Deshui Liu, Lindan Shi, Chenggui Han, Jialin Yu, Dawei Li, Yongliang Zhang

**Affiliations:** State Key Laboratory of Agro-Biotechnology, College of Biological Sciences, China Agricultural University, Beijing, China; Virginia Tech, United States of America

## Abstract

*Nicotiana benthamiana* is the most widely-used experimental host in plant virology. The recent release of the draft genome sequence for *N. benthamiana* consolidates its role as a model for plant–pathogen interactions. Quantitative real-time PCR (qPCR) is commonly employed for quantitative gene expression analysis. For valid qPCR analysis, accurate normalisation of gene expression against an appropriate internal control is required. Yet there has been little systematic investigation of reference gene stability in *N. benthamiana* under conditions of viral infections. In this study, the expression profiles of 16 commonly used housekeeping genes (*GAPDH, 18S, EF1α, SAMD, L23, UK, PP2A, APR, UBI3, SAND, ACT, TUB, GBP, F-BOX, PPR* and *TIP41*) were determined in *N. benthamiana* and those with acceptable expression levels were further selected for transcript stability analysis by qPCR of complementary DNA prepared from *N. benthamiana* leaf tissue infected with one of five RNA plant viruses (*Tobacco necrosis virus A*, *Beet black scorch virus*, *Beet necrotic yellow vein virus*, *Barley stripe mosaic virus* and *Potato virus X*). Gene stability was analysed in parallel by three commonly-used dedicated algorithms: geNorm, NormFinder and BestKeeper. Statistical analysis revealed that the *PP2A, F-BOX* and *L23* genes were the most stable overall, and that the combination of these three genes was sufficient for accurate normalisation. In addition, the suitability of *PP2A, F-BOX* and *L23* as reference genes was illustrated by expression-level analysis of *AGO2* and *RdR6* in virus-infected *N. benthamiana* leaves. This is the first study to systematically examine and evaluate the stability of different reference genes in *N. benthamiana*. Our results not only provide researchers studying these viruses a shortlist of potential housekeeping genes to use as normalisers for qPCR experiments, but should also guide the selection of appropriate reference genes for gene expression studies of *N. benthamiana* under other biotic and abiotic stress conditions.

## Introduction

*Nicotiana benthamiana* has become a very important subject for the study of host–pathogen interactions, particularly those involving plant viruses. Many laboratories throughout the world undertake their research work on *N. benthamiana* and the number of published reports involving *N. benthamiana* has increased significantly over the past score years (Figure S1) [1], [2]. It is most likely that *N. benthamiana* has been adopted as a model plant primarily due to its unparalleled susceptibility to viruses, which is associated with the naturally occurring mutation in the RNA-dependent RNA polymerase gene, *NbRdRP1m* [3]. Three major technical advances have contributed to increased utilisation of *N. benthamiana* in the field of plant biology: plant virus-based expression vector systems, virus-induced gene silencing (VIGS) and agro-infiltration.

Plant virus-based transient expression systems provide attractive alternatives for the production of antibodies, vaccines, growth factors and many other proteins of pharmaceutical importance, because they offer several potential advantages compared to the traditional transgenic approach, including easy manipulation, high yield and fast manufacturing [1], [4], [5], [6]. In most cases, *N. benthamiana* has been used as the experimental host for the development of plant virus-based expression systems, such as *Tobacco mosaic virus*-based MagnICON vectors, *Cowpea mosaic virus*-based expression systems, and others [5], [7], [8].

Along with the advances in plant virus-based transient expressions, another recent major technological breakthrough for plant viral amplicon is now known as virus-induced gene silencing (VIGS), which dramatically accelerated the process by which plant molecular biologists are able to unravel the functions of genes in a large number of plant species [1], [9], [10], [11], [12], [13]. Furthermore, *N. benthamiana* has provided a critical platform for the construction and application of VIGS vectors. Almost without exception, *N. benthamiana* is also the preferred host plant for testing the effectiveness of currently available VIGS vectors in silencing marker genes (e.g. *NbPDS*), followed by procedures that extend the VIGS to economically important plants [9], [10]. In addition, VIGS mediates the homology-based post-transcriptional degradation of selected plant RNA, leading to a loss-of-function phenotype [14]. Senthil-Kumar *et al*. (2007) found that tobacco rattle virus (TRV)-mediated VIGS can be performed on a wide range of solanaceous plant species and that heterologous gene sequences from distantly-related plant species can be used to silence their respective orthologs in the VIGS-efficient plant, *N. benthamiana* [1], [15]. In 2012, scientists at the Boyce Thompson Institute for Plant Research (BTI) released a draft sequence of the *N. benthamiana* genome [1], which has made it easier to identify orthologous genes of tomato, potato and other plant species. All these advancements have transformed *N. benthamiana* into a powerful reverse-genetics system for rapid identification of genes of interest from a wide range of plant species [1], [16], [17], [18].

The third technological advancement that has served to popularise *N. benthamiana* as a research model was agro-infiltration [1], [2], [19]. Firstly, whole genomes of many plant viruses, such as the viral vectors mentioned above, were cloned into binary vectors and delivered into plants via agro-infiltration, which is clearly superior to inoculation with *in vitro* transcripts of full-length viral cDNA [20], [21], [22]. Secondly, agro-infiltration works exceptionally well in *N. benthamiana* but poorly in other plants, including *Arabidopsis thaliana* [23]. The transgenic *N. benthamiana* line 16C has been instrumental in elucidating the mechanism of RNA silencing and for the identification of virus-encoded suppressors of silencing [24], [25], [26], [27]. Proteins of interest were often expressed as fusions to auto-fluorescent proteins by agro-infiltrating the leaf tissue of *N. benthamiana* to study their subcellular localisation [28], [29]. Additionally, sufficient leaf tissue of *N. benthamiana* can be infiltrated to permit small-scale protein purification needed for biochemical analysis [30], and co-infiltration of *N. benthamiana* is also widely used to identify protein–protein interactions through bimolecular fluorescence complementation (BiFC), pull-down assays and co-immunoprecipitation (Co-IP) [31], [32], [33], [34], [35].

Taken together, the methods described above, or combinations thereof, illustrate the importance of *N. benthamiana* as an indispensable research model in plant virology and its increasing usefulness in many aspects of plant biology [1], [2].

To date, a VIGS-cDNA library has been constructed for large-scale gene function analyses of *N. benthamiana* [16], [18], [36], [37], [38], [39]. Comparative analysis of transcriptomic and proteomic responses of *N. benthamiana*–virus interactions have been performed [40], [41], [42], [43] and a microarray platform suitable for RNA profiling of virus-infected *N. benthamiana* (the Nb-array) is also available [2]. Although a large number of differently-expressed genes have been identified using the methods described above, more accurate quantitative analysis of target genes, in order to further confirm their transcription levels within particular contexts, is absolutely necessary before taking the next step.

Quantitative real-time reverse transcription–polymerase chain reaction (qRT–PCR) is the most common method for either characterising or confirming gene expression patterns, and comparing mRNA levels across different sample populations, due to its high sensitivity, specificity, accuracy and reproducibility [44], [45], [46]. The most prominent problem with quantitative mRNA analysis, however, is the selection of an appropriate control gene for accurately normalising gene expression data [47], [48]. An ideal endogenous control gene, also called a reference or housekeeping gene, is one that is stably expressed within the samples to be compared, regardless of tissue differences, experimental conditions or treatments. The use of reference genes that are neither valid nor stable can have a significant impact on the results obtained, possibly leading to erroneous conclusions [47], [49], [50]. Thus, the choice of an appropriate housekeeping gene for normalisation purposes is a prerequisite in qRT–PCR experiments. This is of particular importance when using qRT–PCR to measure viral and cellular gene transcription levels in the context of viral infections, since viruses can significantly interfere with host cell pathways, the components of which are often encoded by traditional housekeeping genes [51]. Moreover, different viruses are likely to manipulate different transcription pathways, and the stability of a selected reference gene often fluctuates according to variations in virus and host species, as well as the timing of a virus infection and the cell type infected [52], [53], [54], [55].

Many studies have been undertaken to determine reliable reference genes in plant cells across various plant species, developmental stages, and root and shoot abiotic and biotic stresses [56]. For *N. benthamiana*. Rotenberg *et al*. evaluated the suitability of three genes, *ubi3*, *EF-1* and actin as internal controls for quantifying gene expression when using TRV-based VIGS vectors, although *EF-1* and *ubi3* were found to be least variable among rTRV-infected and rTRV::tPDS-infected *N. benthamiana*, both genes were induced two- to three-fold by TRV infection compared to the non-infected plants, thus a reference gene that is not altered by TRV infection hadn’t been identified [57]. As *N. benthamiana* had become the most popular platform for the study of virus–host interactions and accurate quantification of functional genes involved in the interaction is critical [1], [2], there is now an urgent need to systematically analyse the reference gene stability for qRT–PCR on *N. benthamiana* plants in the context of viral infection. In this study, we aimed to evaluate different reference genes for qRT–PCR gene expression experiments on cDNA from *N. benthamiana* leaf tissue, which was either uninfected or infected with one of five RNA plant viruses. We selected 16 candidate reference genes, some of which were commonly used as normalisation factors in qRT–PCR analysis, such as *GAPDH*, *18S*, *EF1α*, *ACT* and *TUB* [56], and other genes showed a high expression stability in plant species under viral infection or other experimental settings. The suitability of those candidate reference genes with acceptable expression levels was then statistically evaluated with geNorm [58], NormFinder [59] and BestKeeper algorithms [60]. Finally, the effects of using these internal control genes with good rankings in the statistical analyses were further assessed through relative quantification of *AGO2* and *RdR6* gene expression levels in virus-infected *N. benthamiana*.

## Results

### Assessment of Target Specificity and Amplification Efficiencies in qRT–PCR Reactions

The stabilities of 16 *N. benthamiana* candidate internal controls were tested under different biotic stress conditions, as determined by the infection of five pathogenic viruses. The five viruses belonged to four different genera: *Necrovirus* (TNV-A and BBSV), *Benyvirus* (BNYVV), *Hordeivirus* (BSMV) and *Potexvirus* (PVX). Infections were confirmed by Western blotting (Figure S2). Using the primer pairs designed for each of the 16 candidate reference genes (Table 1), gene-specific amplification was confirmed by the appearance of a single peak in melting curve analyses following qRT–PCR (Figure S3). Agarose gel electrophoresis of the amplicons produced single fragments of the expected size (83–149 bp) in all cases (Figure S4A). No band was detected in the negative control, demonstrating the absence of genomic DNA contamination (Figure S4B). Sequencing analyses showed that all genes were 100% identical to those deposited in public databases (unpublished data). Amplification efficiencies ranged from 90.4% to 104.7% (Table 1), which is well within the acceptable range of 90–105%, as suggested in a qRT–PCR optimisation guide from Bio-Rad Laboratories, Inc. (http://www.gene-quantification.de/real-time-pcr-guide-bio-rad.pdf). Furthermore, the standard curves demonstrated good linear relationships (R^2^>0.980) between the cycle threshold (Ct) values and the log-transformed copy numbers for all tested reference genes (Table 1), confirming the suitability of the primer pairs and target sequences in the qRT–PCR-based quantification.

**Table 1:** Primers and amplicon characteristics for candidate internal control genes, *AGO2* and *RdR6.*

Gene symbol	Gene name	Accession number	Primer sequences (5′-3′) forward/reverse	A (bp)	Tm (°C)	E (%)	R^2^
*ACT*	Actin	AY594294 (At2g37620)	TCCTGATGGGCAAGTGATTAC/TTGTATGTGGTCTCGTGGATTC	114	82.0	95.7	0.985
*PPR*	Pentatricopeptide repeat containing protein	GO602734 (At1g62930)	ATGAGGGTCCATTTGAGTGAC/AGGCTGATGTTGGAATCTGG	107	79.0	95.9	0.990
*TIP41*	TIP41-like protein	CK289168 (At4g34270)	ACGAGGATGAATTGGCCGATAA/CCAGAAACGCAGCAATAGGAAC	92	–	–	–
*TUB*	β-Tubulin	EH371301 (At5g12250)	CAAGATGCTACTGCAGACGAG/CTGGAAGTTGTGGTTTTGGC	126	77.0	104.7	0.982
*UK*	Uridylate kinase	EH363935 (At5g26667)	CTAGGAGTATATTGGAAGAGCG/AAAGATACATCGCCTTGCTGAA	107	77.5	100.0	0.993
*18S*	18S rRNA	TC23401 (AtMg01390)	GCAAGACCGAAACTCAAAGG/TGTTCATATGTCAAGGGCTGG	107	82.0	95.6	0.986
*APR*	Adenine phosphoribosyltransferase like	TC21069 (At1g27450)	CATCAGTGTCGTTGCAGGTATT/GCAACTTCTTGGGTTTCCTCAT	108	79.5	94.3	0.991
*EF1α*	Elongation factor 1-α	TC19582 (At5g60390)	AGCTTTACCTCCCAAGTCATC/AGAACGCCTGTCAATCTTGG	116	81.0	93.8	0.994
*GAPDH*	Glyceraldehyde 3-phosphate dehydrogenase	TC21175 (At1g12900)	AGCTCAAGGGAATTCTCGATG/AACCTTAACCATGTCATCTCCC	125	81.0	99.1	0.983
*GBP*	GTP binding protein	TC20872 (At5g59840)	GGAACTGGATTCGCAACATAGA/GACCCTTGGAAGTTGGCACAGC	114	80.0	92.5	0.982
*L23*	60S ribosomal protein	TC19271 (At2g39460)	AAGGATGCCGTGAAGAAGATGT/GCATCGTAGTCAGGAGTCAACC	110	78.5	95.3	0.994
*PP2A*	Protein phosphatase 2A	TC21939 (At1g13320)	GACCCTGATGTTGATGTTCGCT/GAGGGATTTGAAGAGAGATTTC	123	78.0	98.2	0.995
*SAMD*	S-adenosyl methionine decarboxylase	TC18571 (At3g02470)	GCAAGGGTGGTTCCATTGTCTA/GGCCCTCAAGACACTACTCCTT	129	80.0	90.4	0.981
*UBI3*	Ubiquitin3	TC20187 (At5g03240)	AATGTGAAAGCCAAGATCCAAG/CGGAGGCGGAGCACGAGATGAA	149	80.0	91.0	0.991
*F-BOX*	F-box protein	Niben.v0.3.Ctg24993647 (At5g15710)	GGCACTCACAAACGTCTATTTC/ACCTGGGAGGCATCCTGCTTAT	127	79.0	100.8	0.993
*SAND*	Sand family protein	Niben.v0.3.Ctg25188435 (At2g28390)	ACCACCAACACCTATGAATGCT/CAGTCTCGCCTCATCTGGGTCA	83	79.0	93.4	0.995
*AGO2*	Putative Argonaute-2 protein	EH367034 (At1g31280)	CATTTGAACCTCCTTTCTATCGAC/CATACCTCTAGAAGTGAGGATCAC	121	78.0	91.1	0.982
*RdR6*	Putative RNA-dependent RNA polymerase SDE1	AY722008 (At3g49500)	TTCAGGAATGTCTTCGAGCG/AGTGATCTAGCAACCCAATGAG	134	78.5	93.3	0.982

List of *N. benthamiana* selected candidate reference genes: symbol, name, accession numbers, designed qRT–PCR primer sequences, length of the amplified fragment (A), melting temperature (Tm), efficiency of PCR amplification as calculated by CFX Manager Software (E), and the coefficient of determination (R^2^). Selected *N. benthamiana* candidate reference gene accessions are shown as identifiers of GenBank database, DFCI *N. benthamiana* Gene Index (TC) [91] and Sol Genomics Network (Niben) [92]. Their corresponding homologs in *A. thaliana* are also shown (in brackets).

### Expression Levels of Candidate References Genes

**Figure 1:**
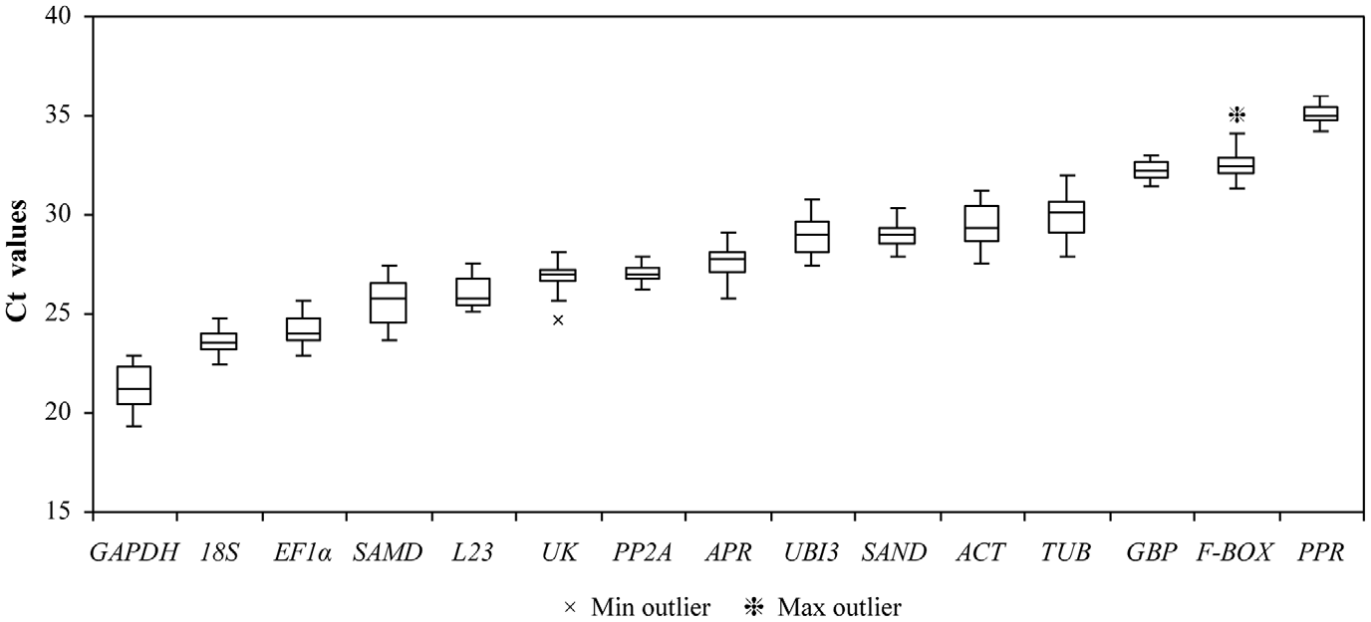
Expression levels of candidate reference genes in healthy and virus-infected leaf tissue samples of *Nicotiana benthamiana*. Values are given as the cycle threshold (Ct, mean of triplicate samples) and are inversely proportional to the amount of template. Global expression levels of the different genes tested are shown as the 25^th^ and 75^th^ quartiles (horizontal lines), median (central horizontal line) and whiskers. Whiskers go either from the minimal to maximal value or, if the distance from the first quartile to the minimum value is more than 1.5 times the interquartile range (IQR), from the smallest value included within the IQR to the first quartile. Outliers, values smaller (Min) or larger (Max) than 1.5 times the IQR, are indicated. Genes are in order from the most (lower Ct, on the left) to the least abundantly expressed (higher Ct, on the right).

In order to provide an overview of the relative abundance of the 16 candidate reference genes in *N. benthamiana*, we determined Ct values for all of these genes except *TIP41* from five different viral infections using three biological and three technical replicates (n = 54 for each gene). From the graph, the median Ct values of the mRNAs that were selected as candidates ranged from 21.22 (*GAPDH*) to 34.97 (*PPR*) (Figure 1), which respectively represented the highest and lowest accumulation levels in virus-infected *N. benthamiana* leaf tissue. In a preliminary experiment, *TIP41* was excluded from further analyses due to very low transcript accumulation (>35 cycles). In this study, we used 40 cycles of amplification for the qRT–PCR; therefore, Ct values of 35 were late to amplify (i.e. late call) and considered unreliable. Consequently, *PPR* was also removed from further analysis, despite its narrow range of Ct values (Table S1). This is consistent with a previous study, in which *TIP41* and *PPR* also showed the lowest expression levels in virus-infected *A*. *thaliana* [52]. The exclusion of *TIP41* and *PPR* resulted in a final list of 14 candidate reference genes for further transcript stability analysis.

The range of Ct values under different treatments indicated a considerable variability among the 14 candidate reference genes. The least amount of variation in gene expression across all 54 tested samples occurred in *GBP* and *PP2A* (<2 cycles), while *TUB* was the most variable (>4 cycles) (Table S1). A simple comparison of the raw Ct values, however, is not sufficient for evaluating expression stability of the candidate reference genes. Consequently, a sophisticated statistical analysis, as described below, was needed to provide a more accurate assessment of the reference genes.

### Analysis of Candidate Reference Gene Stability

To further evaluate the expression stability of the candidate reference genes, we applied three commonly-used algorithms to calculate expression stabilities individually, namely geNorm [58], NormFinder [59] and BestKeeper [60].

### geNorm Analysis

**Figure 2:**
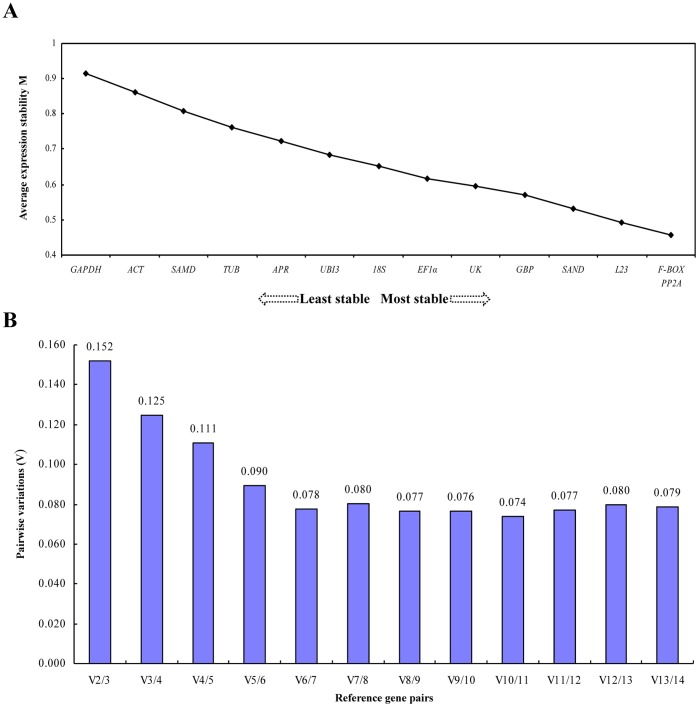
Expression stability of the candidate reference genes analysed by geNorm. (A) Average expression stability values (M) following stepwise exclusion of the least stable reference genes across all treatment groups. A lower M value indicates more stable gene expression. (B) The optimal number of reference genes required for effective qRT–PCR data normalisation. The pairwise variation (V_n_/V_n+1_) was analysed between the normalisation factors NF_n_ and NF_n+1_ using geNorm software to determine whether inclusion of an additional reference gene adds to the stability of the normalisation factor.

The raw Ct values were transformed into quantities for relative comparison. Average gene expression stability (M value) of the 14 candidate reference genes was calculated with the geNorm applet, and all candidates were ranked based on the M values (Figure 2). A lower value of average expression stability (M) indicated a more stable expression. Pairwise variation (V) was implemented to calculate the optimal number of genes to include when performing normalisation using multiple reference genes [58]. The 14 selected candidate genes all achieved high expression stability criterion, with M <0.92, which is well below the default limit of 1.5 suggested by geNorm (Figure 2A).

We also determined the optimal number of reference genes, according to the pairwise variation value (V_n/n+1_ value) (Figure 2B). The most stably expressed genes of the pool (*F-BOX, PP2A* and *L23*) produced an optimal normalisation of the qRT–PCR data, and the addition of a fourth, less stable normalisation factor (*SAND*) did not significantly increase the statistical reliability of this calculation (Figure 2B). Indeed, the V2/3 value of *F-BOX* and *PP2A* (i.e. pairwise variation when the number of normalisation factors is increased from two to three) was 0.152 (Figure 2B), which was very close to the proposed cut-off value of V = 0.15 [58]. The inclusion of the third housekeeping gene (*L23*), in addition to *F-BOX* and *PP2A*, may have reduced the V value to below the cut-off threshold of 0.15, which would have suggested that the top three reference genes (*F-BOX*, *PP2A* and *L23*) would be adequate in our qRT–PCR normalisation of different viral infections, and an additional reference gene was not required.

### NormFinder Analysis

NormFinder is another Microsoft Excel-based Visual Basic application that assigns stability values to single candidate reference genes. The NormFinder algorithm uses a model-based approach for the estimation of expression variation among candidate genes, taking into account intra- and intergroup variation for normalisation factor calculations and avoiding misinterpretations caused by artificial selection of co-regulated genes [59].

**Figure 3:**
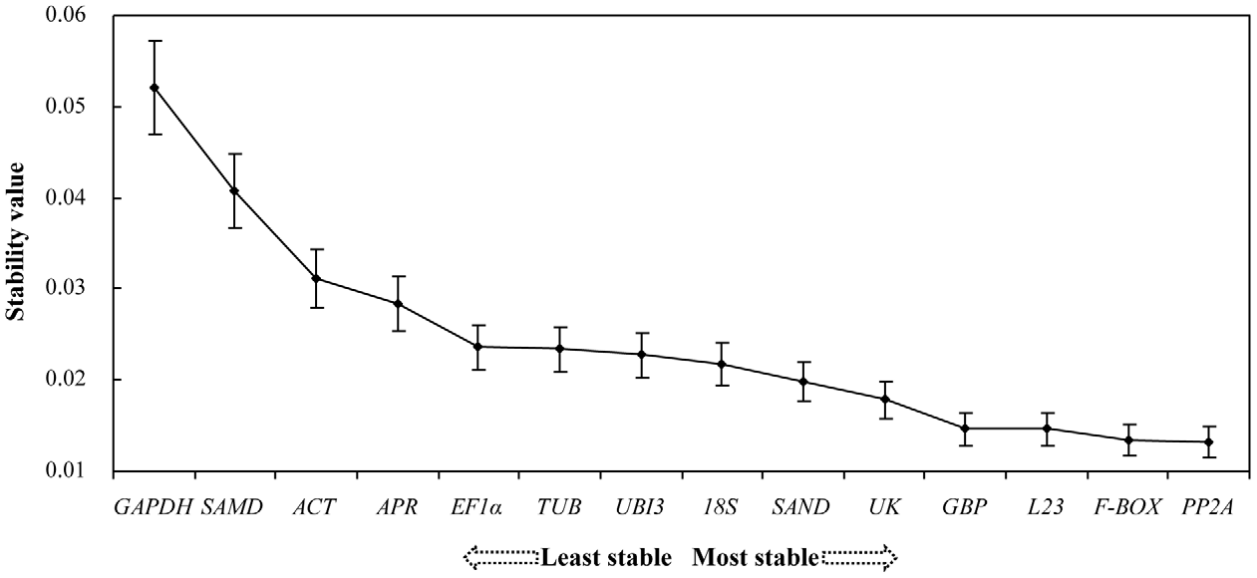
Expression stability of the candidate reference genes analysed by NormFinder. Error bars represent ± standard errors.

We applied a NormFinder analysis to our data (Figure 3). In doing so, the entire ranking order of genes was repeated, and we found limited differences between the geNorm and NormFinder results. *PP2A*, *F-BOX* and *L23* still occupied the three top positions, demonstrating higher stability in virus-infected leaf tissues, with stability values ranging from 0.0132 to 0.0146, whereas *GAPDH*, *ACT* and *SAMD* were again shown to be the least stable genes, with stability values ranging from 0.0312 to 0.0521 (Table 2). The ranking of moderately stable genes according to NormFinder differed slightly from that calculated by geNorm. This was as expected, given that the two statistical algorithms are distinct. Overall, the results of the NormFinder analysis were highly consistent with those obtained from geNorm.

**Table 2:** Ranking of the candidate reference genes according to their stability value using geNorm, NormFinder and BestKeeper analyses.

	geNorm		NormFinder		BestKeeper*	
Gene name	Stability value	Ranking order	Stability value	Ranking order	Stability value	Ranking order
*F-BOX*	0.457	1	0.0135	2	0.731	4
*PP2A*	0.457	1	0.0132	1	0.748	3
*L23*	0.493	3	0.0146	3	0.833	2
*SAND*	0.530	4	0.0198	6	0.656	6
*GBP*	0.571	5	0.0147	4	0.555	8
*UK*	0.594	6	0.0178	5	0.560	7
*EF1α*	0.615	7	0.0236	10	0.858	1
*18S*	0.651	8	0.0217	7	0.444	9
*UBI3*	0.685	9	0.0227	8	0.695	5
*APR*	0.722	10	0.0283	11	0.401	10
*TUB*	0.762	11	0.0234	9	–	11
*SAMD*	0.809	12	0.0407	13	–	13
*ACT*	0.863	13	0.0312	12	–	12
*GAPDH*	0.915	14	0.0521	14	–	14

* In the BestKeeper analysis, the genes of *TUB*, *SAMD*, *ACT* and *GAPDH* were rank-ordered according to their CV [% Ct] and SD [± Ct].

### BestKeeper Analysis

BestKeeper evaluates gene expression stability for all individual reference genes based on three variables: standard deviation (SD), coefficient of correlation (r) and percentage covariance (CV). All reference genes are combined into an index (BestKeeper). The correlation between each reference gene and the index is calculated, based on individual Ct values, as the geometric mean of a number of candidate reference genes. Reference genes with SD values >1 are considered inconsistent and should be excluded [60].

Initial analysis of the data using variations (SD [± Ct] and CV [%Ct]) for all the candidate reference genes showed that all candidate reference genes had SD values <1, except for *GAPDH* (SD = 1), indicating that most were suitable to be considered for selection as reference genes (Table 3).

**Table 3:** Descriptive statistics of the fourteen candidate reference genes based on their cycle threshold (Ct) values as calculated by BestKeeper.

Ctor	Reference gene													
	*EF1α*	*L23*	*PP2A*	*F-BOX*	*UBI3*	*SAND*	*UK*	*GBP*	*18S*	*APR*	*TUB*	*ACT*	*SAMD*	*GAPDH*
Ranking	1	2	3	4	5	6	7	8	9	10	11	12	13	14
N	54	54	54	54	54	54	54	54	54	54	54	54	54	54
Geo Mean [Ct]	24.19	25.99	27.05	32.51	28.93	29.01	26.96	32.27	23.54	27.60	29.97	29.42	25.55	21.20
ar Mean [Ct]	24.20	26.00	27.05	32.51	28.95	29.02	26.97	32.27	23.55	27.61	29.99	29.44	25.57	21.23
Min [Ct]	22.86	25.09	26.21	31.34	27.47	27.85	25.71	31.50	22.41	25.80	27.88	27.53	23.66	19.29
Max [Ct]	25.65	27.51	27.85	34.12	30.82	30.31	28.12	33.01	24.80	29.07	31.96	31.24	27.46	22.92
**SD [± Ct]**	**0.60**	**0.61**	**0.36**	**0.45**	**0.74**	**0.49**	**0.39**	**0.39**	**0.49**	**0.60**	**0.79**	**0.84**	**0.94**	**1.00**
**CV [% Ct]**	**2.49**	**2.34**	**1.34**	**1.39**	**2.55**	**1.69**	**1.46**	**1.20**	**2.07**	**2.16**	**2.62**	**2.84**	**3.68**	**4.70**
Min [x-fold]	−2.36	−1.86	−1.79	−2.25	−2.76	−2.23	−2.38	−1.70	−2.13	−3.47	−4.26	−3.71	−3.71	−3.76
Max [x-fold]	2.58	2.87	1.74	3.05	3.69	2.46	2.23	1.67	2.32	2.78	3.97	3.53	3.75	3.29
SD [± x-fold]	1.50	1.50	1.27	1.35	1.64	1.39	1.30	1.29	1.39	1.49	1.72	1.79	1.92	2.00
**coeff. of corr. [r]**	**0.858**	**0.833**	**0.748**	**0.731**	**0.695**	**0.656**	**0.560**	**0.555**	**0.444**	**0.401**	–	–	–	–
*p*-value	0.001	0.001	0.001	0.001	0.001	0.001	0.001	0.001	0.001	0.003	–	–	–	–

n: number of samples; [Ct]: cycle threshold; Geo Mean [Ct]: geometric mean of Ct value; ar Mean [Ct]: arithmetic mean of Ct value; Min and Max [Ct]: extreme values of Ct; Min and Max [x-fold]: extreme values of expression levels expressed as absolute x-fold over, or under, coefficient; Std dev [±x-fold]: standard deviation of the absolute regulation coefficients; SD [±Ct]: standard deviation of Ct value; CV [%Ct]: coefficient of variation expressed as the percentage of the Ct value. The correlation between each candidate reference gene and the BestKeeper index was calculated using the Pearson correlation coefficient [r] and the *p*-value [60]. The data used in evaluating the stability of reference genes are indicated in bold.

As was the case with geNorm and NormFinder, BestKeeper also ranked *TUB*, *ACT* and *SAMD* as the least stable reference genes after *GAPDH*, with relatively high CV and SD values (>2.5 and >0.75, respectively) (Table 3). Therefore, *GAPDH*, *SAMD*, *ACT* and *TUB* were excluded from further analysis, leaving 10 reference genes for subsequent assessment using pairwise correlation and regression analysis.

Compared with the geNorm and NormFinder results, BestKeeper found small differences in the ranking of the most stable genes (Table 3). *EF1α* was the most stable gene, with the highest correlation coefficient (r = 0.858). This was followed by *L23*, *PP2A* and *F-BOX* (0.731< r <0.833; *p* value = 0.001), which were the three most stable genes identified by geNorm and NormFinder. This difference may have occurred because the statistical algorithms used by these three methods were distinct. geNorm detects the two reference genes whose expression ratios show the least variation from those of the other tested genes, NormFinder takes intra- and intergroup variation into account for normalisation factor calculations, whereas BestKeeper considers the least variation of a single reference gene.

In summary, regardless of the ranking order (Table 2), a comparison of the three methods (geNorm, NormFinder and BestKeeper) suggests that *PP2A*, *F-BOX* and *L23* could be the most suitable reference genes for normalising mRNA levels within the context of the different viral infections tested in this study. Conversely, *APR*, *TUB*, *SAMD*, *ACT* and *GAPDH* showed relatively low expression stability in leaf tissues of *N. benthamiana* during viral infections.

### *APR* and *EF1α* are the Most Suitable Reference Genes for Normalising the Transcripts from TRV-infected *N. benthamiana*

The TRV-based VIGS vector is a powerful reverse genetics tool for functional gene analysis in plants [10], [16]. A previous report showed that *UBI3* and *EF1α* were suitable for comparing rTRV-infected to rTRV::tPDS-infected plants based on BestKeeper software analysis, but a reference gene unaltered by TRV infection in non-infected plants was not identified [61]. To further test possible benefits of using TRV-based VIGS, the 10 most stable reference genes, as determined above, were analysed using qRT–PCR to evaluate their stability in *N. benthamiana* under infection with TRV. We did not comprehensively analyse gene expression stability under TRV infection in conjunction with the results from the other five viruses described above, since TRV infection can lead to severe fluctuations in the expression levels of some internal controls, as has been previously noted [61].

Both geNorm and NormFinder analyses revealed that *APR* and *EF1α* were the most stable reference genes in *N. benthamiana* during TRV infection, and that these two genes were also sufficient for accurate normalisation in TRV-infected leaf tissue, with a V value (0.116) lower than the default cut-off point of 0.15 (Figure S5A, B, C). *UBI3*, however, was ranked lowest in our study (Figure S5A, C), which was inconsistent with a previous report [61]. This result was not surprising because the statistical algorithms and primers used in our study were different from those used previously [61], [62]. BestKeeper analysis of TRV-infected *N. benthamiana* showed a slightly different ranking order from that obtained by geNorm and NormFinder (Figure S5D), which was also not unexpected, as discussed above.

In summary, we are the first to identify, *APR* and *EF1α* as the most suitable reference genes for normalising transcripts from TRV-infected *N. benthamiana*.

### Validation of Reference Genes for Determining the Expression of *AGO2* and *RdR6* in Response to Viral Infection

To further evaluate the reliability of the top three reference genes (*F-BOX*, *PP2A* and *L23*), we selected *AGO2* and *RdR6* for additional qRT–PCR analysis, since they play crucial roles in RNA-based antiviral immunity [63]. *F-BOX*, *PP2A* and *L23* were used in combination as reference genes for expression normalisation. In our previous study, Northern blot analysis showed that the transcription level of *AGO2* could be up-regulated during BBSV infection (Figure S6); therefore, BBSV-infected *N. benthamiana* plants could serve as a positive control.

Relative quantification analysis revealed a significant up-regulation of the *AGO2* expression in all virus-infected *N. benthamiana* leaf tissue. Among them, BNYVV and TNV-A infected leaves presented the highest *AGO2* expression levels compared with mock-inoculated plants, with 12.5- and 11.0-fold increases, respectively. Next came BBSV and BSMV, which caused 7.7- and 7.3-fold changes in *AGO2* expression, respectively.

**Figure 4:**
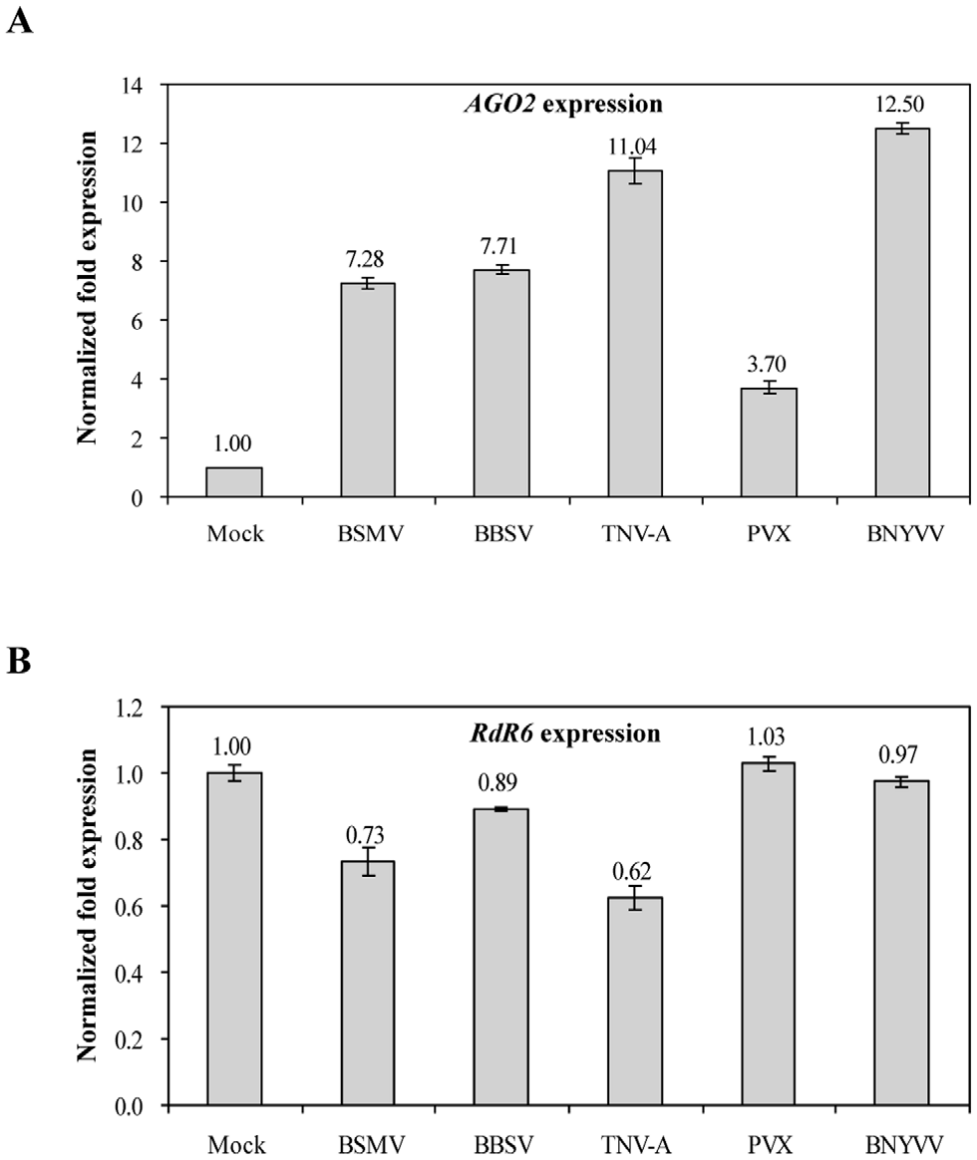
Relative quantification of *AGO2* and *RdR6* expression in *Nicotiana benthamiana* infected with different viruses, with *PP2A*, *F-BOX* and *L23* in combination as reference genes. Error bars represent mean standard error calculated from three biological replicates. Normalisation factors were calculated as the geometric mean of the expression levels of the three most stable reference genes (*F-BOX*, *PP2A* and *L23*) as determined by geNorm analysis. A control mock-inoculated sample was used as the calibrator ( = 1).

qRT–PCR analysis of the *AGO2* expression level in BBSV-infected *N. benthamiana* matched that of Northern blotting (Figure S6). PVX also induced a 3.7-fold increase of the *AGO2* mRNA level in the leaves of *N. benthamiana* (Figure 4A). These results suggest that *AGO2* plays a general role in mediating RNA silencing-based defences against plant viruses, and also confirm the suitability of *F-BOX*, *PP2A* and *L23* as internal controls.

In contrast to the expression pattern of *AGO2*, there was no obvious decrease in *RdR6* expression between virus-infected and mock-inoculated groups, with relative quantification values ranging from 0.62 to 1.02. BBSV, BSMV and TNV-A triggered a weak down-regulation of *RdR6* in *N. benthamiana*, while the PVX and BNYVV-infected group showed almost no change (Figure 4B). The expression profile of *RdR6* in this study was similar to that from virus-infected *A*. *thaliana*, as previously described [52].

## Discussion

### Quantitative Real-time PCR

Quantitative reverse transcription–polymerase chain reaction (qRT–PCR) is one of the best methods available for determining changes in gene expression [45]. Prior to analysis of target gene expression, it is essential to select an appropriate normalisation strategy to control for non-specific variation between samples. Reference genes that have stable expressions at different biological and physiological states can be effectively used for normalisation [62]. However, there is no universal reference gene that can be stably expressed under all experimental conditions. A well-tested housekeeping gene demonstrating significant expression stability in one plant species or tissue type may not show the same stability if used in different experimental situations, species or tissues [49], [64]. Thus, it is essential that prior validation of all reference genes should be performed to confirm their expression stabilities either under certain experimental conditions or for different tissues/cells, in order to prevent inaccurate data interpretation and subsequent false conclusions [46], [49], [50].

### The Necessity and Urgency of Validating Reference Genes for Gene Expression Studies in *Nicotiana benthamiana*

*Nicotiana benthamiana* possess the unique trait of being susceptible to a vast number of plant viruses, and consequently, has become a cornerstone for the study of virus–host interactions in plants. Meanwhile, *N. benthamiana* is also rapidly gaining popularity in general plant biology due to its superiority as a platform for virus-induced gene silencing, transient protein expression, protein localisation and so on [1], [2]. However, despite being an indispensable research model, there is limited information on the expression stability of reference genes in *N. benthamiana* under specific conditions. Schmidt *et al*. studied 8 potential reference genes for normalisation of qRT-PCR in *Nicotiana tabacum* during development and abiotic stress [58], however, *N. tabacum* differs from *N. benthamiana* after all, plus that the external conditions used for examine the stability of the candidate reference genes were different from that of our study, so it is improper to apply the stable reference genes obtained in *N. tabacum* for *N. benthamiana* mechanically. In addition, although Rotenberg found suitable reference genes for target gene normalisation when using TRV-VIGS vector in *N. benthamiana* [57], the selected internal controls could only be used for comparing the dynamics of gene expression level between TRV-VIGS-infected and TRV-infected *N. benthamiana* and were not appropriate for normalising gene expression between TRV-infected and non-inoculated plants.

In our study, we systematically evaluated the accumulation and stability of 14 candidate reference genes in *N. benthamiana* subjected to five diverse plant viral infections. In theory, the diversity of these selected viruses should be favourable for the screening of suitable reference genes in *N. benthamiana*. Reference genes identified in our study could be used not only for gene expression quantification when probing gene function by VIGS (eg. TRV, PVX and BSMV-based VIGS vectors) in *N. benthamiana* [16], [37], [65], but also for the study of these genes involved in the interaction between *N. benthamiana* and a particular virus, thus providing convenience and preferred alternative for quantifying gene expression in *N. benthamiana* during viral infection.

### Reference Gene Stability in *Nicotiana benthamiana* during Viral Infections

We analysed the expression of 14 candidate reference genes using three different software packages: geNorm, NormFinder and BestKeeper. Analysis using geNorm was an easy method to determine the optimal number of stable housekeepinggenes for accurate normalisation [58], whereas NormFinder and BestKeeper were used to assess the quality of the ranking obtained by geNorm [59], [60]. The top three reference gene positions in virus-infected samples as defined by geNorm were similar to those determined by NormFinder, yet slightly different from those of the BestKeeper analysis. *EF1α* was ranked first by BestKeeper, but had relative high CV [%Ct] and SD [±Ct] values. The second to fourth positions in the BestKeeper ranking matched the three most stable reference genes according to the geNorm and NormFinder analyses. This variation wasn’t surprising, since the three software packages use different calculation algorithms. Comparable results have also been encountered and discussed in many previous studies [53], [66], [67].

Taken together, the three software packages showed that *PP2A*, *F-BOX* and *L23* were overall the most stable reference genes. Our results corroborate a paper recently published by Lilly *et al*. [52], which tested reference genes for normalising transcripts from virus-infected *A. thaliana*. They found that *F-BOX* and *PP2A* demonstrated the most stable transcript accumulation. Similar results for the *Manihot esculenta* Crantz were observed by Moreno *et al*., whereby the expression patterns of *PP2A*, along with those of *UBQ10* and *GBP*, appeared to be the most stable in assorted *Cassava brown streak virus*-infected tissues and cassava varieties [55]. In addition, *F-BOX* was found to be one of the most stably expressed genes in citrus under diverse experimental conditions, such as tissues versus organs, and various biotic stresses (virus, fungi, or bacteria) [66]. *L23* demonstrated the highest expression stability in our study, which is consistent with the results observed in *N. tabacum* during its development and under abiotic stress [58]. However, it should be pointed out that there is no universal control gene that is expressed at a constant level under all conditions and in all tissues, and *PP2A*, *F-BOX* and *L23* are no exception. For example, *F-BOX* was revealed as the worse gene by all three approaches for qPCR in *Fagopyrum esculentum* based on transcriptome sequence data [68], *PP2A* was not a good choice for normalization of gene expression levels during berry development [69], and the expression level of *L23* was found to be up-regulated in *Tortula ruralis* under rehydration [70]. Despite the unsatisfactory performance of *PP2A*, *F-BOX* and *L23* in other plants under particular conditions, we believe that these three reference genes have the potential to be extensively used as internal controls for target gene normalisation in the study of virus–host interaction.

All three software packages ranked *SAND*, *GBP*, *UK*, *18S* and *UBI3* in middle positions for all samples of virus-infected *N. benthamiana*. The novel reference gene, *SAND*, was not ideal for analysing gene expression in virus-infected *N. benthamiana*, yet it was the recommended reference gene for normalisation of transcripts from citrus and virus-infected *A. thaliana* [52], [66]. *GBP* showed very stable expression in *Cassava brown streak virus*-infected cassava as described above [55]. *UK* and *UBI3* were stably expressed throughout virus-infected tissues of tomato plants [53]. It also should be noted that the CV [%Ct] and SD [±Ct] values were very low for *UK* and *GBP* in the BestKeeper analysis of this study.

*18S* and *EF1α* are commonly-used housekeeping genes in qRT–PCR experiments. Previously, *EF1α* has been reported to be stably expressed in potato during biotic and abiotic stress [71] and *18S* is suitable for normalisation in *Barley yellow dwarf virus*-infected cereals [54]. In this study, however, the performance of *EF1α* and *18S* was unsatisfactory, even though *EF1α* was ranked top by the BestKeeper analysis. Our results were consistent with earlier studies in *Cicer arietinum* and virus-infected tomato, both of which showed considerable regulation for *18S* and *EF1α* [53], [72]. Taken together, our data again demonstrates that the widely-used reference genes *18S* and *EF1α*, may not be the optimal choices for testing transcript abundance in *N. benthamiana* during viral infection.

*APR* and *SAMD* were found to be the most suitable reference genes in *Brassica rapa* and *Brachypodium distachyon*, respectively [73], [74]. However, in our study both displayed high variation in their expression levels, possibly because they implement certain functions during viral infections. These results suggest that it is necessary to collect as much gene expression data as possible in different organisms, organs and experimental conditions, in order to avoid selecting a reference gene which is itself involved in the regulation of the gene of interest under its particular conditions. For example, the DNAJ-like protein, a traditional housekeeping gene used in previous studies [75], was excluded from our analysis during the initial experimental design because its expression is associated with replication of plant virus [76]. This may also explain why *UBI3* exhibited poor performance during our study.

*GAPDH*, *ACT* and *TUB* have traditionally been the three most widely-used reference genes for transcript expression analysis in various plant species [54], [56], [66]. However, their suitability as internal standards has recently come into question, especially due to their potential regulation in a wide variety of physiological states [52], [67], [71]. In this study, analyses using all three software packages ranked *TUB*, *ACT* and *GAPDH* in the bottom positions. *GAPDH*, the lowest ranked gene, showed the highest expression variability of all samples. Our results again suggest plant virologists should be cautious when using *TUB*, *ACT* or *GAPDH* as internal controls for accurate normalisation of a target gene in virus-infected *N. benthamiana*.

It is also worth noting that the samples of BBSV-infected leaf tissue derived from a temperature controlled greenhouse at 18°C, a relatively low temperature compared to the 24°C used for the other four viruses. That being said, all the samples were analysed together without distinction, so it is reasonable to speculate that the stable reference genes identified in our study may be suitable for normalising gene expression data in *N. benthamiana* under low-temperature stress. These results suggest a benefit for further quantitative gene expression analysis in *N. benthamiana*.

In summary, the results presented above further emphasise the necessity of identifying and validating a reference gene for a given plant under a particular set of experimental conditions prior to its use for normalisation, as there is no universal reference gene suitable for all experimental situations [50], [56], [77]. More importantly, we believe our study will provide researchers with a convenient and reliable data resource when undertaking gene expression studies in *N. benthamiana*.

### Normalisation to a Single Reference Gene or Multiple Reference Genes

Many previous reports have stated the importance of choosing the correct internal controls in their experiments, through comparison of the expression levels of target genes normalised by the most- and least-stable reference genes. They further highlight that incorrect use of reference genes that lack validation can introduce bias in an analysis (i.e., over- and underestimation) and lead to misinterpretation of data [50], [52], [53], [62], [66]. Furthermore, they found patterns of target gene expression to be similar, whether two (or more) of their most stable reference genes were used either individually or in combination. This suggests that one suitable, verified reference gene may be sufficient for normalising data to roughly approximate the expression of a gene of interest [78], [79]. In our study, for example, the expression patterns of *AGO2* and *RdR6* showed similar trends to those of *PP2A*, *F-BOX* and *L23* as internal controls either singly or in combination (Figure 4 and Figure S7), which further indicated that *PP2A*, *F-BOX* and *L23* were suitable as reference genes. However, increasing the number of reference genes for normalisation will improve analytical accuracy. Consequently, two or more reliable reference genes should be used in parallel as internal standards when normalising subtle but significant variations among different samples, despite the fact this is expensive and time-consuming [58], [62], [67].

In the current study, based on V ≤0.15, three reference genes were sufficient for the normalisation of qRT–PCR data from virus-infected *N. benthamiana*. One of the factors that may have made it more difficult for us to achieve V ≤0.15 was the large number of samples and treatments tested, an issue consistent with previous observations [62]. Datasets containing small numbers of samples and treatments have tended to require fewer reference genes for accurate normalisation than larger datasets (e.g., >50 samples; 2–4 treatments). In our study, for example, when the expression stability was analysed separately for each sample treated with a particular virus, it would be easy to obtain the optimal reference genes from among the 14 candidates, and each individual viral infection could be normalised by just two reference genes, whose V values were all lower than the geNorm threshold of 0.15 (unpublished data). In addition, it should also be noted that the proposed 0.15 V value was not an absolute cut-off, as emphasised previously [58], [62], [78]. Our results indicated that combining the two most stable reference genes (*PP2A* and *F-BOX*) gave a V value (0.152) close to 0.15, implying that the use of these two reference genes was sufficient for the normalisation of qRT–PCR data in virus-infected *N. benthamiana*.

Although *APR* and *EF1α* were considered to be optimal reference genes when analysing samples from TRV-infected *N. benthamiana* individually by different algorithms, it didn’t contradict the combined results of *PP2A*, *F-BOX* and *L23* as suitable reference genes across all other virus-infected *N. benthamiana* samples, because the ranking of the reference gene stability was often not uniform when considering data from all the samples versus from each sample, which had been observed in previous reports [75], [80].

Overall, all of the tested reference genes showed relatively high stability with low average expression stability M values less than 0.92, which is far below the default limit of M ≤1.5, evaluation of all expression data from five different viruses-treated samples revealed that *PP2A*, *F-BOX* and *L23* were the most stably expressed genes, suggesting that these may be suitable reference genes for analyses of gene expression in *N. benthamiana* infected with a wide variety of viruses. Our results are a valuable resource for others who seek accurate normalisation of gene expression in experiments on *N. benthamiana* under conditions of viral infection.

### Expression of *AGO2* and *RdR6* in Response to Viral Infection

To further validate the applicability of the screened reference genes, we assessed the expression profile of *AGO2* and *RdR6* genes in virus-infected *N. benthamiana* leaf tissue. *AGO2* and *RdR6* play important roles in RNA silencing-based antiviral defence [63]. Recent studies have shown that the down-regulation of RdR6 in *N. benthamiana* leads to superinfection with PVX and *Plum pox virus*, while no such effect is observed for TRV or *Tobacco mosaic virus* [81], [82]. A similar phenomenon also occurs in the Arabidopsis *AGO2* mutants infected with *Turnip crinkle virus* or *Cucumber mosaic virus* [83]. Moreover, Jaubert *et al*. found that *AGO2* mediated RNA-silencing antiviral defences against PVX in Arabidopsis sp., whereas Scholthof *et al*. concluded the same in *N. benthamiana* during *Tomato bushy stunt virus* infection [84], [85].

In this study, we normalised our potential genes of interest, *AGO2* and *RdR6*, using the normalisation factor generated for the three most stable candidate reference genes, *PP2A*, *F-BOX*, and *L23*. Results showed that *AGO2* expression was evidently up-regulated in virus-infected leaf tissue when compared with the control group, suggesting a more general role for *AGO2* in plant innate immune responses. RdR6 showed a different expression pattern from that of *AGO2*: only three viruses caused a slight down-regulation of *RdR6*. The *RdR6* expression profile in our study was consistent with that in virus-infected Arabidopsis sp. [52]. In addition, Ren *et al.* found that the *OsRDR6* mRNA levels in *Rice dwarf virus*-infected plants decreased to nearly 34% that in non-infected plants [86]. As such, expression of *RdR6* during virus–host interactions is likely associated with pathological traits of a given virus.

In summary, our results further confirm that the three most stable reference genes identified in our study (*PP2A*, *F-BOX* and *L23*) could be used for accurate normalisation of a target gene in virus-infected *N. benthamiana*.

### Conclusions

To the best of our knowledge, this article describes the first attempt to validate a set of commonly-used candidate reference genes, in *N. benthamiana*, that can be used for the normalisation of gene expression analysis using qRT–PCR. We identified 14 reference genes that were suitable for the normalisation of qRT–PCR data, which were obtained from *N. benthamiana* leaf samples subjected to five different viral infections. Evaluations using geNorm, NormFinder and BestKeeper identified the three most suitable reference genes in *N. benthamiana* as *PP2A*, *F-BOX* and *L23*. The least suitable reference gene was *GAPDH*, which may be unsuitable in future *N. benthamiana* studies.

Our results not only provide researchers interested in these viruses with a shortlist of potential housekeeping genes to use as normalisers for qRT–PCR experiments, but also should guide the selection of appropriate reference genes for gene expression studies in *N. benthamiana* under conditions other than those tested here, or in other plant species under similar treatment conditions.

## Materials and Methods

### Virus Inoculation and Sample Preparation

Seedlings of *N. benthamiana* were grown in a glasshouse and were infected with one of five viruses at 4 weeks of age. Virus inocula were first prepared in *N. benthamiana* as follows: BBSV and TNV-A^C^ were mechanically inoculated into *N. benthamiana* with previously described *in vitro* transcripts [87], [88]; BSMV and PVX were agro-inoculated into *N. benthamiana* according to published methods [65]; and BNYVV was propagated on *N. benthamiana*, and its total RNA was extracted from symptomatic leaves to be used as the inoculum [89]. At 7–14 dpi, systemic leaves that had typical symptoms of the corresponding viruses were sampled. These samples were ground in 20 vol. 0.1 M potassium phosphate (K_2_HPO_4_) buffer (pH 7.4) containing carborundum (as an abrasive). Mechanical inoculations were then undertaken using the sap from systemically infected *N. benthamiana* leaf tissue for the test. Simultaneously, mock-inoculated plants without infectious homogenate were created as controls.

All the *N. benthamiana* plants, except those inoculated with BBSV, were maintained in a controlled environmental climate chamber at 24±0.5°C with a photoperiod of 14-hours light (∼75 µmol/m^2^/s) and 10-hours dark. Plants infected by BBSV, together with four mock-inoculated controls, were grown at 18°C, since relatively low temperatures is required for BBSV to establish systemic infection in *N. benthamiana* [90]. At least 12 plants were inoculated for each virus. On days 7–14, typical symptoms appeared in the upper leaves and the infection was further confirmed by Western blotting using virus-specific antiserum (Figure S2). For each of the five viruses, three biological replicates were collected and subjected to RNA extraction. Each replicate consisted of upper leaf tissue pooled from four *N. benthamiana* plants.

### Selection of Candidate Reference Genes

Sixteen genes commonly used as internal controls in previous studies [52], [53], [58], [73], were selected for investigation in order to identify the most stably expressed reference gene(s) in virus-infected *N. benthamiana* (Table 1). Specifically, the *N. benthamiana* orthologs of Arabidopsis sp. *EF1α*, *18S*, *GAPDH*, *L23*, *APR*, *PP2A*, *UBI3*, *GBP* and *SAMD* nucleotide sequences were obtained by querying the DFCI *N. benthamiana* Gene Index [91] using BLASTN. *Nicotiana benthamiana* nucleotide sequences of *ACT*, *TUB*, *UK*, *TIP41* and *PPR* were available from the GenBank database. For *F-BOX* and *SAND* genes, only a sequence of *A*. *thaliana* was found. Based on the recently released *N. benthamiana* whole genome sequence, a BLAST against the SOL Genomics Network database allowed us to identify the nucleotide sequences of *N. benthamiana* corresponding to *F-BOX* and *SAND* [92].

### Total RNA Isolation and First Strand cDNA Synthesis

Total RNA was extracted from approximately 200 mg of freshly sampled leaf tissue using TRIzol reagent (Invitrogen, Carlsbad, CA, USA), according to the manufacturer’s instructions. Any genomic contamination was removed before cDNA synthesis using RNase-free DNase I (TaKaRa, Dalian, China), and according to the manufacturer’s protocols. Nucleic acid quality was estimated by visual analysis on 1.2% agarose gel electrophoresis, according to standard procedures [93]. RNA concentrations were measured using a Nanodrop ND-1000 spectrophotometer (Nanodrop Technologies, Rockland, DE, USA) and only RNA samples with an *A*260/*A*280 ratio in the range 1.8–2.0 were used, in order to minimise the effects of PCR inhibitors. All RNA samples were stored at −80°C.

The first strand of cDNA was synthesised from 1.5 µg total RNA with the M-MLV reverse transcriptase and oligo (dT)_15_ primer (Promega, Madison, WI, USA) according to user instructions. In brief, total RNA samples were denatured at 95°C for 3 minutes in the presence of 10 pM oligo (dT)_15_ primer and then quickly cooled on ice. M-MLV reverse transcriptase and other reaction components were added to the samples. These were then incubated for 10 minutes at 37°C (primer annealing), followed by 90 minutes at 42°C and finally 10 minutes at 70°C to inactivate the enzyme. Reverse transcription (RT) negative controls, without the inclusion of the reverse transcriptase enzyme, were performed in parallel to test for the presence of genomic DNA contamination in RNA samples. Amplification was then conducted for all genes using RT-PCR, followed by assessment on a 4% agarose gel. No visible amplification was detected in any of the control samples (Figure S4B).

### Primer Design, Verification of Selected Gene Amplicons and Gene-specific PCR Amplification Efficiency

For all genes, qRT–PCR primers were designed using the Primer Premier 5.0 software (Premier Biosoft International, Palo Alto, CA, USA) and Oligo 6 software (Molecular Biology Insights, Inc., Cascade, CO, USA). To ensure maximum specificity and efficiency during PCR amplification, a stringent set of criteria was used for primer design [94]. This included predicted melting temperatures (Tm) of 58–60°C, primer lengths of 20–24 nucleotides, GC contents of 50–60% and PCR amplicon lengths of 80–150 bp. All primers were custom-ordered from a commercial supplier (Invitrogen, Shanghai, China).

To check the specificity of all primers, PCR was performed on cDNA and the size of all PCR products was verified on a 4% agarose gel (Figure S4A). PCR products were then purified using a QIAquick Gel Extraction Kit (Qiagen, Hilden, Germany), according to the manufacturer’s instructions, and cloned into pGEM-T Easy Vector (Promega, Madison, WI, USA), followed by sequencing.

The PCR amplification efficiency of each primer was calculated using the equation: Efficiency% = (10^[-1/slope]^ −1)×100%, according to the MIQE guidelines for qRT–PCR experiments [95]. This was presented using Bio-Rad CFX Manager software (version 1.6) on a standard curve generated from a two-fold dilution series of one sample at five dilution points for three technical replicates.

### Quantitative Real-Time PCR

qRT–PCR was performed in 96-well plates using the CFX96 real-time PCR detection system (Bio-Rad, Hercules, CA, USA). Three different biological replicates for each virus infection (n = 12) were used (i.e. reverse-transcribed RNA extracted from four individual plants were pooled together). All cDNA samples were amplified in triplicate from the same RNA preparation and the mean value was considered (i.e. three technical replicates). Each reaction mixture consisted of 1 µl cDNA, 7 µl SsoFast EvaGreen Supermix (Bio-Rad, Hercules, CA, USA), 1.5 µl (3 pmol/µl) of both forward and reverse primers, and 3 µl PCR-grade water (TaKaRa, Dalian, China), equating to a final volume of 14 µl. The thermal profile of the reaction was an initial denaturation at 95°C for 3 minutes, followed by 40 cycles at 95°C for 10 seconds and 60°C for 10 seconds. This was followed by fluorescence acquisition after each cycle. Finally, a dissociation curve was generated by increasing temperature from 65 to 95°C, in order to verify primer specificity (Figure S3). All samples for each reference gene were run on the same plate to avoid between-run variations. Baseline and Ct values were automatically calculated by the CFX Manage version 1.6 software (Bio-Rad, Hercules, CA, USA) with default parameters.

### Determination of Reference Gene Expression Stability Using GeNorm, NormFinder and BestKeeper

To identify the most appropriate genes in each experimental set, the stability of the mRNA expression of each reference gene was statistically analysed using three different types of Microsoft Excel-based software: geNorm [58], NormFinder [59], and BestKeeper [60]. All three software packages were used according to the manufacturer’s instructions.

For geNorm, the raw Ct values were transformed into the required data input format. The maximum expression level (i.e. the lowest Ct value) of each gene was used as a control and was set = 1. Next, relative expression levels were calculated from Ct values using the formula: 2^−ΔCt^, in which ΔCt = each corresponding Ct value - minimum Ct value. Resultant data were further analysed using the geNorm program to calculate the mean pair-wise variation between an individual gene and all other tested candidate reference genes, the results were shown as expression stability (M). Candidate genes with the lowest M value were considered to be most stable under tested experimental conditions. Stepwise exclusion of the gene with the highest instability (highest M value) was performed and a new M value (average expression stability) for each remaining reference gene is calculated until the two most stable genes were left (Figure 2A). Since these calculations are based on ratios, the final two genes cannot be resolved from each other. NormFinder adopted the same input file format as the geNorm before calculation, while BestKeeper analyses were based on untransformed Ct values.

### Relative Quantification of *AGO2* and *RdR6*

*AGO2* and *RdR6* were chosen as genes of interest. *PP2A*, *F-BOX* and *L23*, the three most stably expressed genes identified by geNorm analysis, were also used in combination as a reference genes for the quantification of *AGO2* and *RdR6* expression. The normalisation factor was calculated as the geometric mean of *PP2A*, *F-BOX* and *L23* for each sample. Mock-inoculated plants (treated with sap from healthy plants) were used as a control and calibrator sample. Relative quantification was performed using the Bio-Rad CFX Manager software (version 1.6), which employs a ΔΔC(t) algorithm with a PCR efficiency correction. Results can be automatically generated at the end of qRT–PCR.

## Supporting Information

Figure S1 **Rapid increase in the number of research publications using** ***N. benthamiana.*** Data were obtained from the WEB OF KNOWLEDGE database on the search term “nicotiana benthamiana”.
(PDF)

Figure S2 **Molecular detection of different viruses in mock and upper un-inoculated leaves (Samples) of** ***N. benthamiana.*** Western blot analysis of *N. benthamiana* leaves to confirm infection by six different RNA plant viruses. Proteins from systemically infected leaves were separated by SDS-PAGE and subject to Western blot using specific antiserum against the coat protein of TNV-A^C^, BBSV, BNYVV, BSMV, PVX and TRV, respectively. The molecular weights of the coat proteins of 6 plant viruses are indicated on the right side of each panel.
(PPT)

Figure S3 **Specificity of qRT–PCR amplification.** Dissociation curves of the 18 amplicons after the qRT–PCR reactions, all showing one peak.
(PPT)

Figure S4 **Analysis of the performance of the designed primers listed in** **Table 1** **by regular RT-PCR reactions.** 4% agarose gel electrophoresis showing specific reverse transcription PCR products of the expected size for each reference gene, *AGO2* and *RdR6*. M represents DNA size marker. (B) RT-PCR control reactions to assay for genomic DNA contamination by using an equivalent amount of total RNA without reverse transcription. No specific bands were detected. DNA markers are to the right (M).
(PPT)

Figure S5 **Analysis of gene expression stability in** ***N. benthamiana*** **during TRV infection.** (A) Average expression stability values (M) of 10 candidate reference genes calculated by geNorm. (B) Pairwise (V) to determine the optimal number of reference genes for normalisation. (C) Expression stability values of the candidate reference genes analysed by NormFinder. (D) The average Ct value of each triplicate reaction was used (without conversion) to analyse the candidate reference genes using BestKeeper. Bold characters indicate the basis for assessment of gene expression stability.
(PPT)

Figure S6 **Northern blot analysis of the** ***AGO2*** **RNA accumulation in systemic leaves of BBSV-infected** ***N. benthamiana.*** “10” and “25” indicated that 10 µg and 25 µg of total RNA were used for Northern blot detection, respectively. Ethidium bromide staining of total RNA is shown below as a loading control.
(PPT)

Figure S7 **The expression profile of** ***AGO2*** **and** ***RdR6*** **responsive to viral infections in** ***N. benthamiana*** **.**(studied by qRT-PCR with *PP2A*, *F-BOX* and *L23* as reference genes, respectively). Error bars represent the mean ± standard deviation for n = 12 (biological triplicate, each with technical triplicate).
(DOC)

Table S1 **Raw Ct data.** The Excel spreadsheet contains the raw Ct data used for statistical analysis in this study.
(XLS)
