# Supplementary material for: Validation of Reference Genes for Gene Expression Studies in Virus-Infected Nicotiana benthamiana Using Quantitative Real-Time PCR
Source: PLoS One. 2012 Sep 28;7(9):e46451. doi: 10.1371/journal.pone.0046451 (PMC3460881; doi:10.1371/journal.pone.0046451)
Supplement: Figure S1 — Rapid increase in the number of research publications using N. benthamiana. Data were obtained from the WEB OF KNOWLEDGE database on the search term “nicotiana benthamiana”. (PDF) [file pone.0046451.s001.pdf]

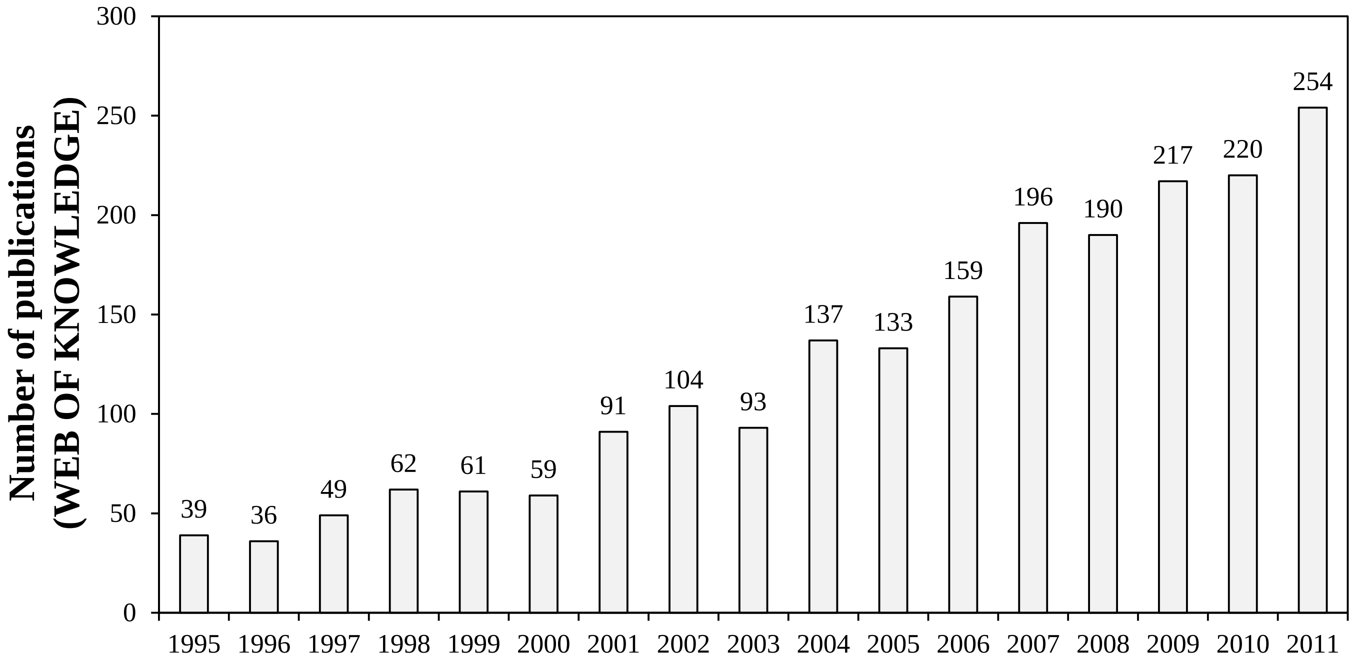

Number of publications
(WEB OF KNOWLEDGE)
0
50
100
150
200
250
300
39
36
49
62
61
59
91
104
93
137
133
159
196
190
217
220
254
1995
1996
1997
1998
1999
2000
2001
2002
2003
2004
2005
2006
2007
2008
2009
2010
2011

Figure S1

Results Analysis

<<Back to previous page

2,100 records. Topic=(nicotiana benthamiana)

Rank the records by this field:	Set display options:	Sort by:
Publication Years<br>Source Titles<br>Subject Areas<br>Web of Science Categories<br>Source Titles	Show the top 500 Results.<br>Minimum record count (threshold): 1	○ Record count<br>⦿ Selected field

Analyze

Use the checkboxes below to view the records. You can choose to view those selected records, or you can exclude them (and view the others).

View Records / Exclude Records	Field: Publication Years	Record Count	% of 2100	Bar Chart
☐	1995	39	1.857 %	
☐	1996	36	1.714 %	
☐	1997	49	2.333 %	
☐	1998	62	2.952 %	
☐	1999	61	2.905 %	
☐	2000	59	2.810 %	
☐	2001	91	4.333 %	
☐	2002	104	4.952 %	
☐	2003	93	4.429 %	
☐	2004	137	6.524 %	
☐	2005	133	6.333 %	
☐	2006	159	7.571 %	
☐	2007	196	9.333 %	
☐	2008	190	9.048 %	
☐	2009	217	10.333 %	
☐	2010	220	10.476 %	
☐	2011	254	12.095 %	
View Records / Exclude Records	Field: Publication Years	Record Count	% of 2100	Bar Chart

Save Analysis Data to File
○ Data rows displayed in table
○ All data rows

Save Analysis Data to File
○ Data rows displayed in table
○ All data rows

© 2012 Thomson Reuters | Terms of Use | *Please give us your feedback on using Web of Knowledge.*
