# Supplementary figures and images for: Validation of Reference Genes for Gene Expression Studies in Virus-Infected Nicotiana benthamiana Using Quantitative Real-Time PCR
Source: PLoS One. 2012 Sep 28;7(9):e46451. doi: 10.1371/journal.pone.0046451 (PMC3460881; doi:10.1371/journal.pone.0046451)

## Slide 1
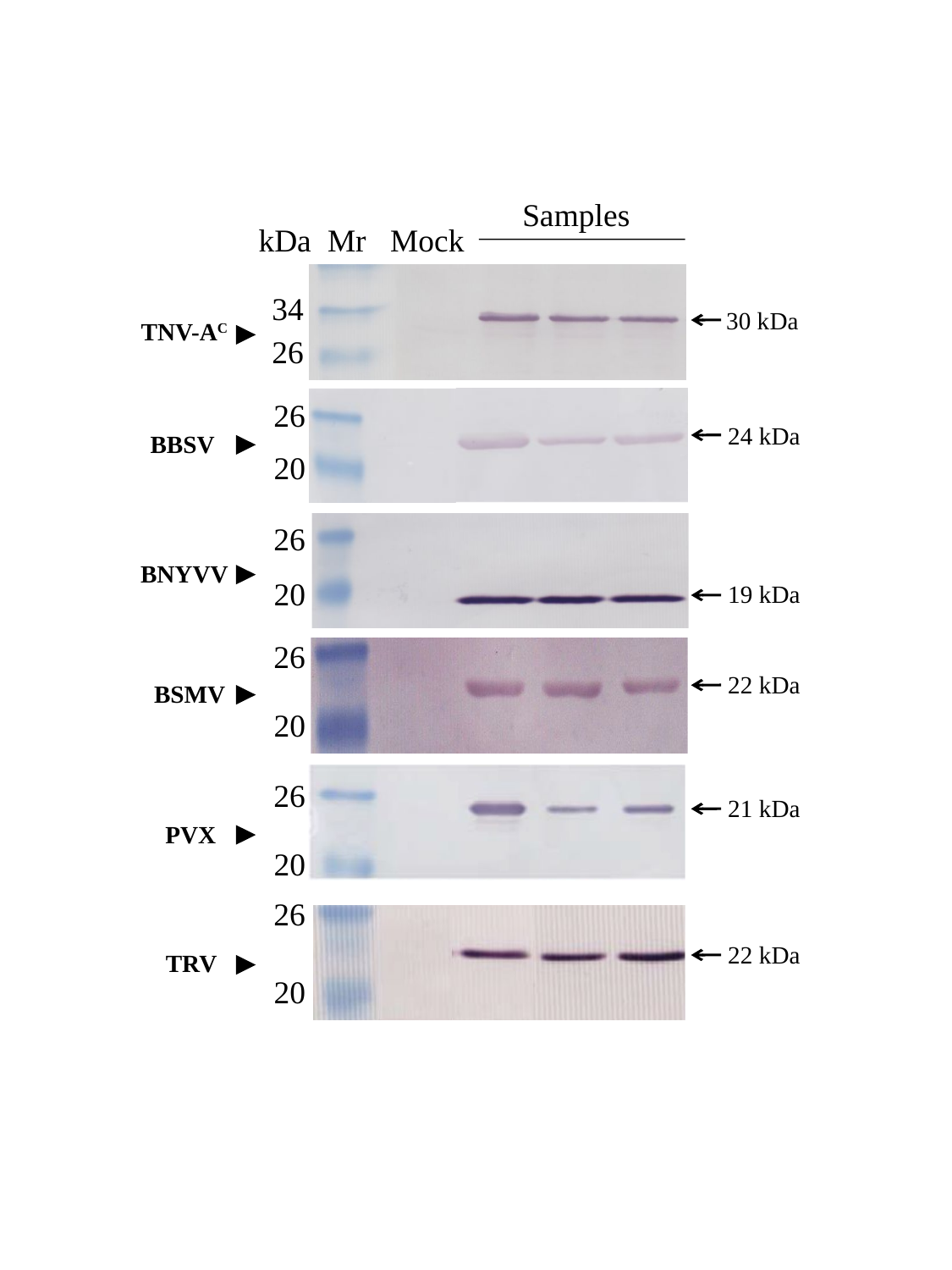

Samples
kDa Mr Mock
34
30 kDa
 TNV-AC
26
26
24 kDa
 BBSV
20
26
 BNYVV
20
19 kDa
26
22 kDa
 BSMV
20
26
21 kDa
 PVX
20
26
22 kDa
 TRV
20

Supplement: Figure S2 — Molecular detection of different viruses in mock and upper un-inoculated leaves (Samples) of N. benthamiana. Western blot analysis of N. benthamiana leaves to confirm infection by six different RNA plant viruses. Proteins from systemically infected leaves were separated by SDS-PAGE and subject to Western blot using specific antiserum against the coat protein of TNV-AC, BBSV, BNYVV, BSMV, PVX and TRV, respectively. The molecular weights of the coat proteins of 6 plant viruses are indicated on the right side of each panel. (PPT) [file pone.0046451.s002.ppt]

## Slide 1
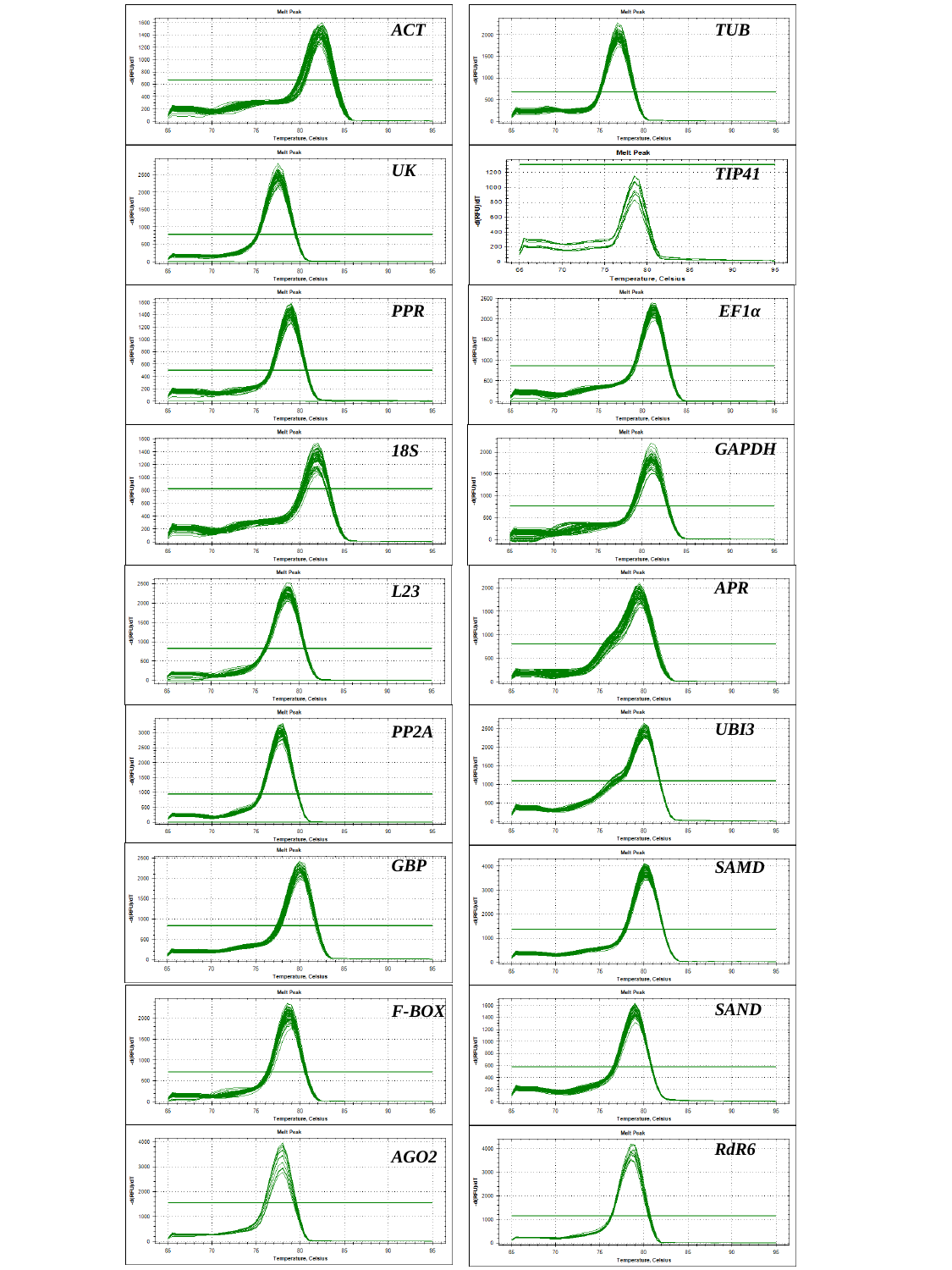

TUB
ACT
UK
TIP41
EF1α
PPR
GAPDH
18S
APR
L23
UBI3
PP2A
GBP
SAMD
SAND
F-BOX
RdR6
AGO2

Supplement: Figure S3 — Specificity of qRT–PCR amplification. Dissociation curves of the 18 amplicons after the qRT–PCR reactions, all showing one peak. (PPT) [file pone.0046451.s003.ppt]

## Slide 1
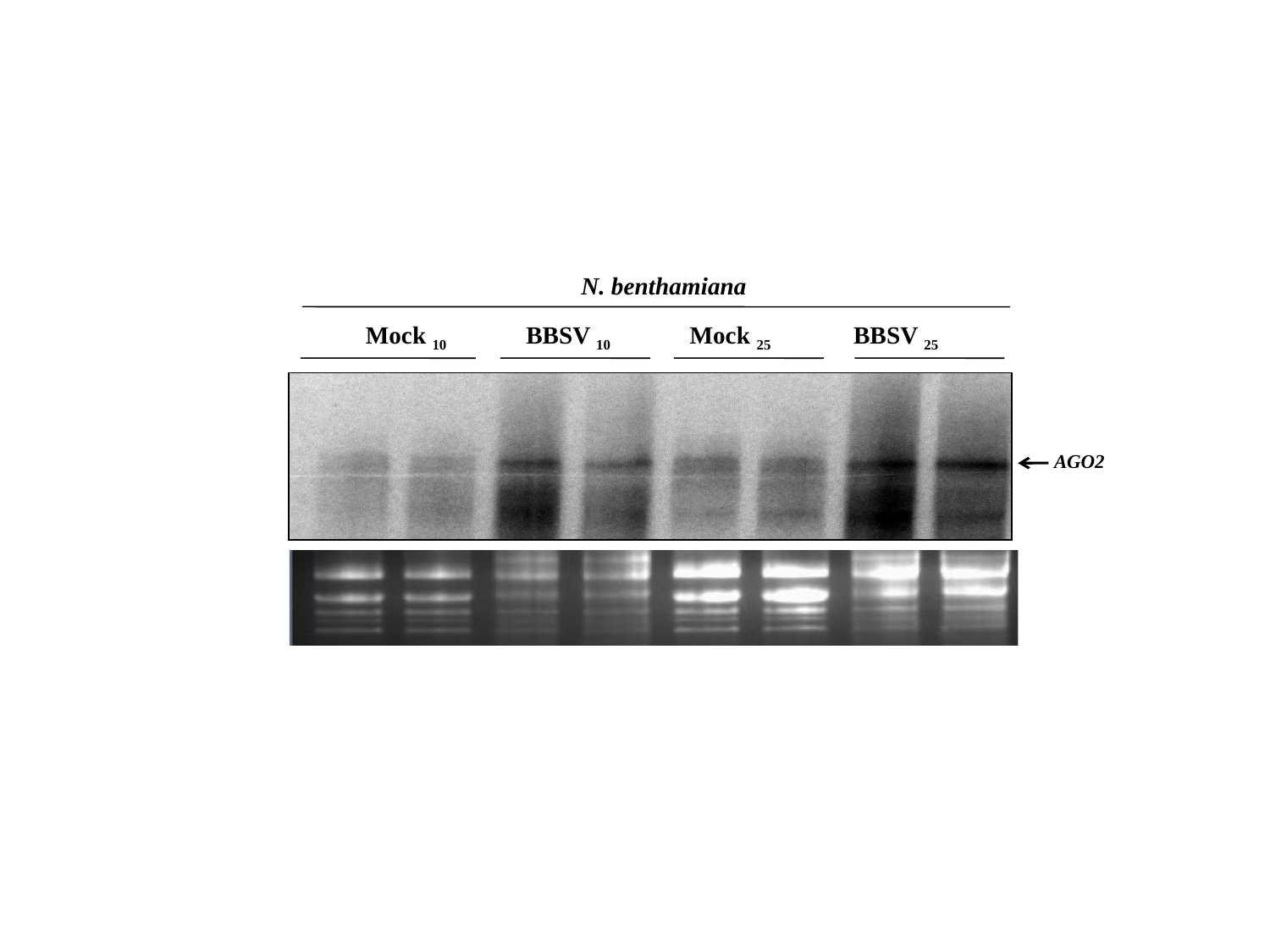

N. benthamiana
 Mock 10 BBSV 10 Mock 25 BBSV 25
AGO2

Supplement: Figure S6 — Northern blot analysis of the AGO2 RNA accumulation in systemic leaves of BBSV-infected N. benthamiana. “10” and “25” indicated that 10 µg and 25 µg of total RNA were used for Northern blot detection, respectively. Ethidium bromide staining of total RNA is shown below as a loading control. (PPT) [file pone.0046451.s006.ppt]
