# Supplementary material for: Validation of Reference Genes for Gene Expression Studies in Virus-Infected Nicotiana benthamiana Using Quantitative Real-Time PCR
Source: PLoS One. 2012 Sep 28;7(9):e46451. doi: 10.1371/journal.pone.0046451 (PMC3460881; doi:10.1371/journal.pone.0046451)
Supplement: Figure S4 — Analysis of the performance of the designed primers listed in Table 1 by regular RT-PCR reactions. 4% agarose gel electrophoresis showing specific reverse transcription PCR products of the expected size for each reference gene, AGO2 and RdR6. M represents DNA size marker. (B) RT-PCR control reactions to assay for genomic DNA contamination by using an equivalent amount of total RNA without reverse transcription. No specific bands were detected. DNA markers are to the right (M). (PPT) [file pone.0046451.s004.ppt]

## Slide 1
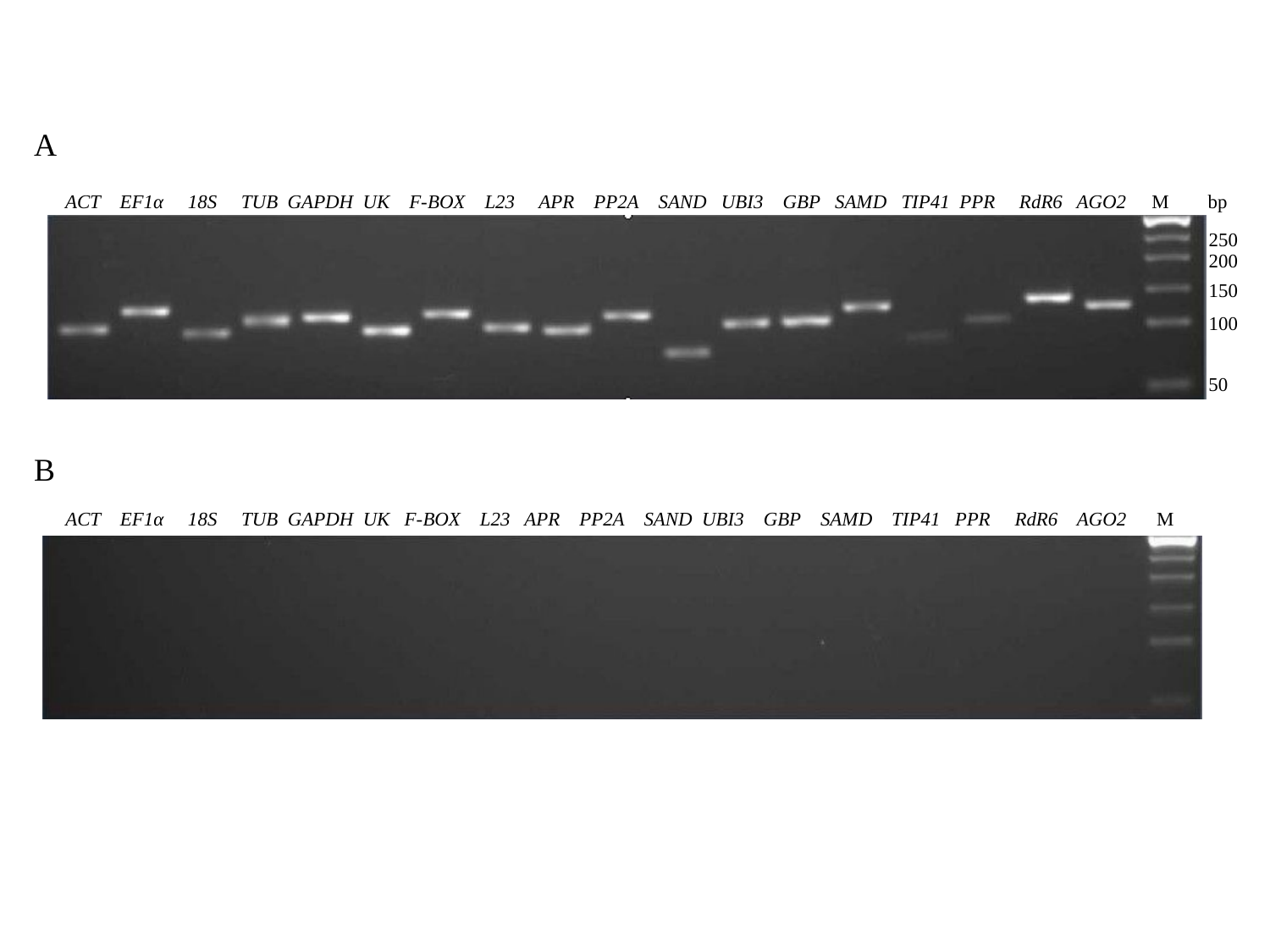

A
ACT EF1α 18S TUB GAPDH UK F-BOX L23 APR PP2A SAND UBI3 GBP SAMD TIP41 PPR RdR6 AGO2 M bp
250
200
150
100
50
B
ACT EF1α 18S TUB GAPDH UK F-BOX L23 APR PP2A SAND UBI3 GBP SAMD TIP41 PPR RdR6 AGO2 M
