# Supplementary material for: Validation of Reference Genes for Gene Expression Studies in Virus-Infected Nicotiana benthamiana Using Quantitative Real-Time PCR
Source: PLoS One. 2012 Sep 28;7(9):e46451. doi: 10.1371/journal.pone.0046451 (PMC3460881; doi:10.1371/journal.pone.0046451)
Supplement: Figure S5 — Analysis of gene expression stability in N. benthamiana during TRV infection. (A) Average expression stability values (M) of 10 candidate reference genes calculated by geNorm. (B) Pairwise (V) to determine the optimal number of reference genes for normalisation. (C) Expression stability values of the candidate reference genes analysed by NormFinder. (D) The average Ct value of each triplicate reaction was used (without conversion) to analyse the candidate reference genes using BestKeeper. Bold characters indicate the basis for assessment of gene expression stability. (PPT) [file pone.0046451.s005.ppt]

## Slide 1
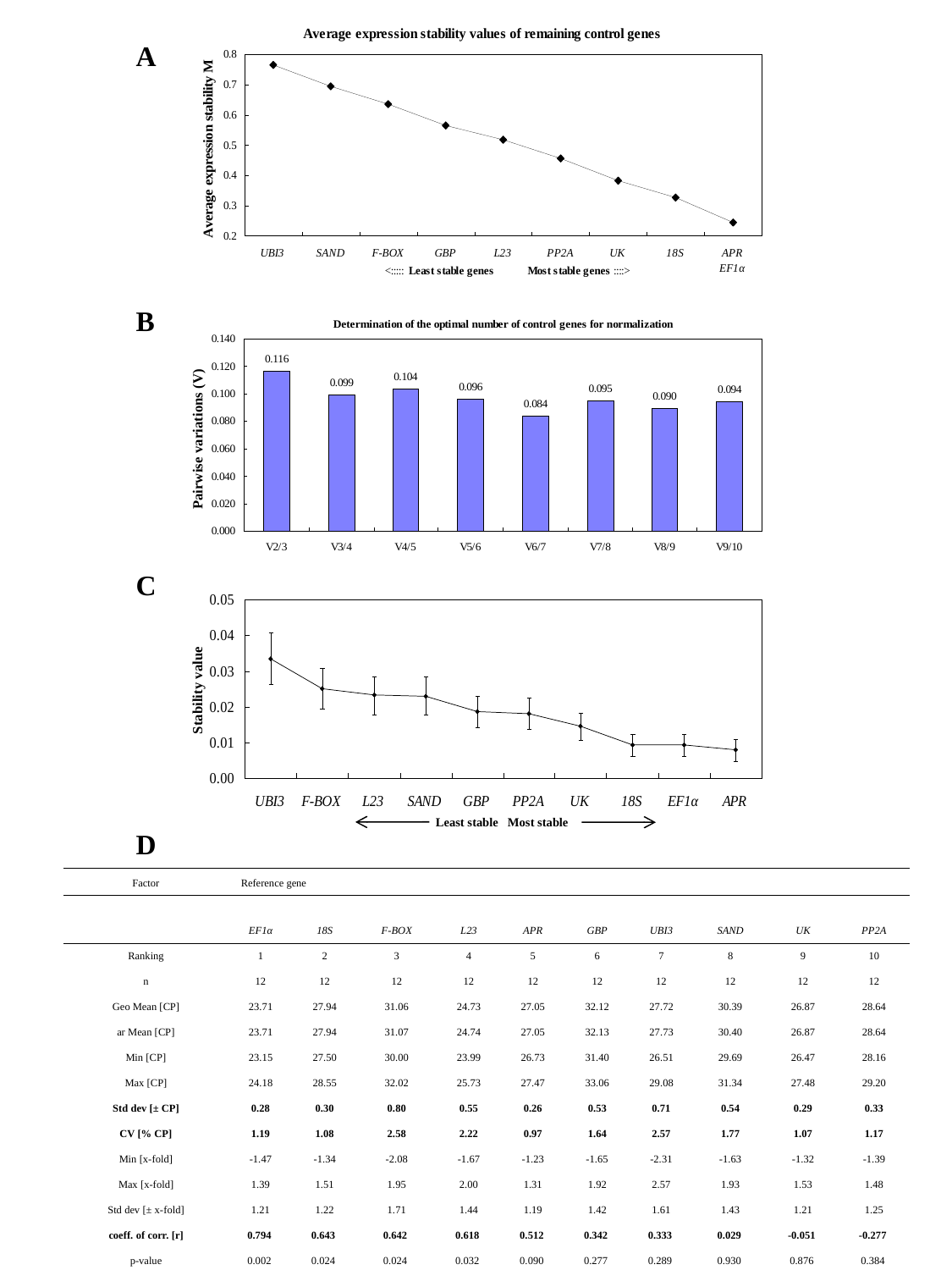

A
B
Pairwise variations (V)
C
Stability value
Least stable Most stable
D
| Factor | Reference gene | | | | | | | | | |
| --- | --- | --- | --- | --- | --- | --- | --- | --- | --- | --- |
| | EF1α | 18S | F-BOX | L23 | APR | GBP | UBI3 | SAND | UK | PP2A |
| Ranking | 1 | 2 | 3 | 4 | 5 | 6 | 7 | 8 | 9 | 10 |
| n | 12 | 12 | 12 | 12 | 12 | 12 | 12 | 12 | 12 | 12 |
| Geo Mean [CP] | 23.71 | 27.94 | 31.06 | 24.73 | 27.05 | 32.12 | 27.72 | 30.39 | 26.87 | 28.64 |
| ar Mean [CP] | 23.71 | 27.94 | 31.07 | 24.74 | 27.05 | 32.13 | 27.73 | 30.40 | 26.87 | 28.64 |
| Min [CP] | 23.15 | 27.50 | 30.00 | 23.99 | 26.73 | 31.40 | 26.51 | 29.69 | 26.47 | 28.16 |
| Max [CP] | 24.18 | 28.55 | 32.02 | 25.73 | 27.47 | 33.06 | 29.08 | 31.34 | 27.48 | 29.20 |
| Std dev [± CP] | 0.28 | 0.30 | 0.80 | 0.55 | 0.26 | 0.53 | 0.71 | 0.54 | 0.29 | 0.33 |
| CV [% CP] | 1.19 | 1.08 | 2.58 | 2.22 | 0.97 | 1.64 | 2.57 | 1.77 | 1.07 | 1.17 |
| Min [x-fold] | -1.47 | -1.34 | -2.08 | -1.67 | -1.23 | -1.65 | -2.31 | -1.63 | -1.32 | -1.39 |
| Max [x-fold] | 1.39 | 1.51 | 1.95 | 2.00 | 1.31 | 1.92 | 2.57 | 1.93 | 1.53 | 1.48 |
| Std dev [± x-fold] | 1.21 | 1.22 | 1.71 | 1.44 | 1.19 | 1.42 | 1.61 | 1.43 | 1.21 | 1.25 |
| coeff. of corr. [r] | 0.794 | 0.643 | 0.642 | 0.618 | 0.512 | 0.342 | 0.333 | 0.029 | -0.051 | -0.277 |
| p-value | 0.002 | 0.024 | 0.024 | 0.032 | 0.090 | 0.277 | 0.289 | 0.930 | 0.876 | 0.384 |
