# Supplementary material for: Validation of Reference Genes for Gene Expression Studies in Virus-Infected Nicotiana benthamiana Using Quantitative Real-Time PCR
Source: PLoS One. 2012 Sep 28;7(9):e46451. doi: 10.1371/journal.pone.0046451 (PMC3460881; doi:10.1371/journal.pone.0046451)
Supplement: Figure S7 — The expression profile of AGO2 and RdR6 responsive to viral infections in N. benthamiana .(studied by qRT-PCR with PP2A, F-BOX and L23 as reference genes, respectively). Error bars represent the mean ± standard deviation for n = 12 (biological triplicate, each with technical triplicate). (DOC) [file pone.0046451.s007.doc]

**Figure S7. The expression profile of *AGO2* and *RdR6* responsive to viral infections in *N. benthamiana*** (studied by qRT-PCR with *PP2A*, *F-BOX* and *L23* as reference genes, respectively). Error bars represent the mean ± standard deviation for *n* = 12 (biological triplicate, each with technical triplicate).

***AGO2 AGO2 AGO2***

***RdR6 RdR6 RdR6***

***PP2A F-BOX L23***
